# Development of a Universal Epitope-Based Influenza Vaccine and Evaluation of Its Effectiveness in Mice

**DOI:** 10.3390/vaccines10040534

**Published:** 2022-03-29

**Authors:** Ramil R. Mintaev, Dina V. Glazkova, Olga V. Orlova, Elena V. Bogoslovskaya, German A. Shipulin

**Affiliations:** 1 Center for Strategic Planning and Management of Medical and Biological Health Risks, Federal State Budgetary Institution, Federal Medical-Biological Agency, 119833 Moscow, Russia; georgin2702@gmail.com (D.V.G.); orlovaov@gmail.com (O.V.O.); lenabo2@mail.ru (E.V.B.); shipgerman@gmail.com (G.A.S.); 2 I. Mechnikov Research Institute of Vaccines and Sera, 105064 Moscow, Russia

**Keywords:** influenza, epitope, MVA, universal vaccine

## Abstract

Vaccination is an effective and economically viable means of protection against the influenza virus, but due to rapid viral evolution, modern seasonal vaccines are not effective enough. Next-generation vaccines are designed to provide protection against a wide range of influenza virus strains, including pandemic variants. In our work, we made an epitope-based universal vaccine, rMVA-k1-k2, against the influenza virus based on the modified vaccinia Ankara (MVA) vector and using our own algorithms to select epitopes from conserved fragments of the NP, M1 and HA proteins of influenza A and B. We show that double immunization protects mice with a 67% or greater efficiency against viral influenza pneumonia when infected with various strains of the H1N1, H2N2, H3N2 and H5N1 subtypes of influenza A. In animals, the level of protection provided by the rMVA-k1-k2 vaccine was comparable to that provided by the universal M001 and MVA-NP+M1 (Invictus) vaccines, which have shown success in clinical trials, against strains of the H1N1 and H3N2 subtypes.

## 1. Introduction

Seasonal influenza affects 5–15% of the human population annually, which affects public health and harms the economies of countries. At the same time, 3–5 million severe cases of the disease and up to 650,000 deaths are recorded every year, and up to 100,000 of these deaths occur in children under 5 years of age [1]. Of the three types of influenza viruses, the most significant pathogens for humans are types A (H1N1 and H3N2 subtypes) and B. Influenza A virus (IAV) also circulates among animals and especially among birds (highly pathogenic viruses of the H5, H7 and H9 subtypes), creating the potential for the emergence of pandemic variants [2,3].

Vaccination is the most effective and economically viable means of protection against seasonal epidemics of the influenza virus. However, the effectiveness of the seasonal vaccines in current use strongly depends on the antigenic profile of the circulating strains coinciding with the variant that was recommended by the WHO for vaccine production. Due to the rapid evolution of the virus, the current strains, presumably prevailing in the next epidemic season, are predicted every year [4]. However, even if the circulating strains coincide with the vaccine, vaccination efficiency does not exceed 60% [5]. This is because seasonal vaccines are aimed at producing antibodies against the immunodominant surface antigens of the virus, which very quickly accumulate mutations that allow them to evade the immune response. In addition, the short-term protective effect of modern influenza vaccines declines significantly after 6 months and after 3 months in the elderly [6].

Thus, there is an obvious need to develop a universal vaccine that can protect against a wide variety of viruses and provide a long-term immune response. Such vaccines should provoke a cross-reactive immune response against a wide range of strains of the same or different subtypes [7].

Several universal vaccines are in different stages of clinical trials, including three epitope vaccines, M001 (BiondVax Pharmaceuticals Ltd., Jerusalem, Israel), Flu-V (hVivo, London, UK) and FP-01.1 (Immune Targeting Systems Ltd., London, UK), as well as two vector vaccines, MVA-NP+M1 (Vaccitech Ltd., Oxford, UK) and ChAdOx1-NP+M1 (University of Oxford, UK), which are based on the conserved internal NP and M1 proteins of the influenza virus [8]. Simultaneously, five consortia are currently engaged in the development of next-generation influenza vaccines only in Europe. Their approaches differ in immunization strategy, antigen composition and delivery method [9].

When a full-sized protein antigen is introduced into the body, a hierarchy of the effectiveness of different epitopes of this antigen arises: immunity is produced more strongly for immunodominant epitopes, but most often they are variable. However, if conserved epitopes are introduced separately (weakly immunogenic epitopes as part of the whole antigen), then in the absence of competition against them, a more effective immune response is produced [10]. Thus, epitope vaccines have a number of advantages because they allow the immune system to focus on conserved epitopes.

The aim of the present work is to develop an epitope-based universal vaccine based on MVA, a compelling vector for antigen delivery due to its safety and immunogenicity in humans, even in those with immunodeficiency, to compare it with previously described candidate analogs of universal vaccines under similar conditions [11,12,13,14], and to estimate the range of protection and immunogenicity of the developed vaccine.

## 2. Materials and Methods

### 2.1. Epitope Selection

The IEDB [15] was used to select immunogenic epitopes from influenza virus. Epitopes were selected from conserved regions of the viral HA, NP and M1 proteins. Protein sequences were extracted from the influenza virus database [16] in April 2019. Entries were obtained for hemagglutinin from type A subtypes H1 (*n* = 13,850) and H3 (*n* = 11,762) and from type B (*n* = 3141); for NP from type A (*n* = 10,292) and type B (*n* = 1413); and for M1 of type A (*n* = 3250).

The sequences of each protein and type/subtype were aligned using the MAFFT program based on the FFT-NS-2 algorithm [17]. Poorly aligned regions were additionally edited using the Jalview program [18]. The Shannon entropy was calculated using a developed script [19] written in Python to estimate the conservation of the aligned column. To calculate the number of types of k amino acid residues that could appear in a given alignment position with a 1/k probability, the formula

k = 2^s^, (1)

was used, where S is the information entropy of this column and is calculated using the formula

S = −∑(q_i_ log q_i_), (2)

where q_i_ is the frequency of occurrence of the ith most common amino acid residue at a given position in a set of aligned sequences. Summarization for entropy was performed for all types of residues found in the aligned column being studied. Thus, the epitope was deemed to be conserved if all of the positions included therein met the criterion of k < 1.3 [20,21].

The selected epitopes were mapped on the surface of the HA protein structure using a molecular visualization program PyMOL [22]. The X-ray diffraction analysis data of hemagglutinin were obtained from the PDB database [23]. The IDs of the records were as follows: for type A subtype H1—5K9O and subtype H3—4KVN and for type B—4FQK.

The frequencies of distribution of allelic variants of major histocompatibility complex (MHC) molecules were taken from the allele frequency net database (AFND) [24].

Influenza virus type A strains A/California/07/2009 (H1 subtype) and A/Victoria/208/2009 (H3 subtype) and type B strain B/Brisbane/60/2008 were used as references to number epitope position.

### 2.2. Cell Lines and Viruses

For the titration of influenza viruses (TCID50 measurements), we used a continuous MDCK cell culture—a culture of dog kidney cells from the cell culture collection of The National Research Center for Epidemiology and Microbiology named after the Honorary Academician N.F. Gamaleya of the Ministry of Health of the Russian Federation. The cells were cultured at 37 °C in MEM medium with 2 mM L-glutamine, 0.1 mM interchangeable amino acids, 1 mM pyruvate salt and 10% fetal bovine serum.

Modified vaccinia Ankara (MVA) was obtained from the American Type Cell Culture Collection (VR-1566, ATCC, Manassas, VA, USA). To amplify the MVA virus, the Syrian hamster fibroblast cell line BHK-21 was used; it was cultured at 37 °C in standard DMEM containing 10% bovine embryonic serum, 4 mm L-glutamine, 1 mM sodium pyruvate and 10 mm HEPES in an atmosphere of 5% CO_2_. Amplification and the calculation of the viral titer (TCID50) were performed according to the protocol described previously [25,26].

Viruses from Table A1 were used to infect mice. Viruses from Table A2 were used to perform the hemagglutination inhibition (HAI) test and neutralization test. We used BSL-3 conditions when working with H2N2 and H5N1 strains.

### 2.3. Construction of Plasmids

The synthesis of nucleotide sequences encoding influenza virus antigens was carried out by TOP Gene Technologies, Inc. (St-Laurent, QC, Canada). The plasmid shuttle vector was obtained earlier in the laboratory [27]. Synthesized inserted antigens k1, k2, k5, NP + M1, hlHA or Multimeric-001 were inserted into the polylinker of the shuttle vector at the NheI and KpnI sites. At the 5′ end of each coding frame, with the exception of hlHA, the Ig k leader sequence was inserted to direct the protein along the secretory pathway [28,29]. A sequence encoding a 6xHis tag was placed at the 3′ end of all constructs. The shuttle vector included a reporter RFP gene controlled by the promoter of the smallpox vaccine virus thymidine kinase (TK) gene needed to select MVA recombinants. In addition, the reporter cassette was flanked with sequence repeats needed for marker gene removal during a second RFP negative selection by means of homologous recombination. Plasmid DNA was propagated in E. coli cells and isolated using a Plasmid Mini Kit (QIAGEN, Hilden, Germany) or Plasmid Maxi Kit (QIAGEN) under sterile conditions. The correctness of the constructs was confirmed by sequencing.

### 2.4. Obtaining Recombinant MVA

The recombinant MVA viruses (rMVA) were obtained, purified and amplified as described previously [27]. In short, BHK-21 cells were infected with the wild-type MVA virus and transfected with a shuttle vector after 90 min, and the medium was replaced after 8 h. After 48 h, the mixture of recombinant and wild-type viruses was collected. Cells growing in a monolayer on a 6-well plate were infected with 10-fold dilutions of the mixture. Two days after infection, the cells were observed under a fluorescence microscope, noting red viral plaques that expressed the RFP gene. The marked plaques were selected and placed in 500 mL of medium for receiving viral suspensions, which were analyzed for insertion using real-time PCR. The procedure was repeated, and samples with the highest percentages of rMVA were taken until a virus containing no wild-type virus impurities was obtained. The absence of wild-type MVA in the recombinant virus was checked using PCR analysis and electrophoresis detection.

### 2.5. Western Blot

Twenty-four hours after transduction with one of the recombinant viruses—rMVA-k1, rMVA-k2, rMVA-k5, rMVA-M001, rMVA-hlHA or rMVA-NP+M1 (multiplicity of infection (MOI) was 2)—BHK-21 cells were collected, washed three times with PBS, resuspended in RIPA lysis buffer containing 1% NP-40 and lysed using ultrasonication. Cell debris was removed by centrifugation (15,000× *g*, 15 min, 4). A PageRuler Plus protein marker (Thermo Fisher Scientific Inc., Waltham, MA, USA) and the cell lysates were subjected to 12% SDS–PAGE (each sample contained 20 micrograms of protein). After electrophoresis under reducing conditions, the proteins were transferred to an Immun-Blot LF PVDF membrane (Bio-Rad Laboratories, Inc., Hercules, CA, USA) using wet electrophoretic transfer. The membranes were blocked in PBS containing 5% skim milk powder and 0.1% Tween 20 and then incubated overnight at +4 °C with antibodies against the 6x-His tag (HIS. H8-HRP, Invitrogen, Carlsbad, CA, USA) at a dilution of 1:20,000 or antibodies against beta-actin (AC-15-HRP, Abcam, Cambridge, UK) at a dilution of 1:50,000. Then, the membranes were washed 3 times in PBS with 0.1% Tween 20. Protein complexes were detected using Clarity Western ECL Substrate reagent (Bio-Rad Laboratories, Inc., Hercules, CA, USA) in accordance with the manufacturer’s recommendations and visualized using X-ray film (Fujifilm, Tokyo, Japan).

### 2.6. Experimental Animals

Nine-day-old chicken embryos obtained from the “Ptichnoe” chicken factory (Ptichnoe, Moscow Region, Russia) were used for their allantoises to obtain virus for infecting animals and to obtain red blood cells for the HAI test.

To study the antigenic and protective properties of experimental versions of the vaccines, 6- to 8-week-old sexually mature female white outbred mice weighing 18–20 g were used. The animals were obtained from the vivarium of the National Center of Biomedical Technologies of the Russian Academy of Sciences, Russia.

The studies were conducted in accordance with the regulatory documents in force in the Russian Federation and took into account the recommendations of the WHO and MG 3.3.2.1758–03 [30,31,32].

### 2.7. Mouse Immunization and Study Design

All animals were immunized intramuscularly with 0.2 mL of a solution containing 4x10E6 infectious MVA particles or the Flu-M influenza inactivated split virion vaccine, which was manufactured by SPb NIIVS FMBA of Russia for the 2019–2020 season and included HA A/Brisbane/02/2018 H1N1 pdm09-like, A/Kansas/14/2017 (H3N2)-like and B/Colorado/06/2017-like, at half the human dose. Animals from the mock group were injected intramuscularly with 0.2 mL of sterile PBS instead of the vaccine preparations on the corresponding days.

In each experiment, the animals were immunized twice: (1) on Day 0, prime vaccination was conducted, (2) and on Day 21, boost vaccination was carried out. On the 21st day after the first immunization and on the 14th day after the second immunization, blood samples were taken from 4 mice from each group for the HAI and neutralization tests. Additionally, on the 14th day after the second immunization, the remaining mice were infected with influenza virus to assess the protective effectiveness after 2 weeks. On the 4th day after infection, the viral titer in the lungs was determined in 3 mice from each group.

### 2.8. HAI Test

The determination of hemagglutinating units in the samples was determined by the standard method of carrying out the HAI test described previously (support protocol 8 in [33]).

### 2.9. The Neutralization Test

The neutralizing titer of the antibodies was measured by setting up a neutralization test as previously described in [34]. In short, starting from 1:10, consecutive two-fold dilutions of the studied mouse sera were mixed in equal volumes with the studied influenza viruses (the dose of the virus constituted 100 TCID50) and added to MDCK cells. To determine the neutralizing titer, the cytopathic effect on MDCK cells was evaluated after 72 h of incubation.

### 2.10. Determination of the Protective Activity of Vaccine Variants

Fourteen days after the second immunization (35 days after the first immunization), the mice were infected intranasally with the studied influenza viruses at a dose of 10 LD50 per mouse. The animals were monitored daily for the next 14 days. The protective activity of the experimental vaccines against viral challenge in a mouse model was evaluated according to three criteria: survival, average lifetime after infection and viral titer in the lungs.

### 2.11. Determination of the Viral Titer in the Lungs of Mice

The viral titer in the lungs of three animals from each group was determined on Day 4 after infection using methods described previously (support protocols 4 and 6 in [33]). In short, the lungs were extracted, homogenized in saline solution under sterile conditions and centrifuged. Then, a series of 10-fold dilutions of the supernatant were added to MDCK cells. The titer was determined based on the cytopathic effect on MDCK cells using the Reed and Muench method and expressed as log_10_ TCID_50_ ([33] support protocol 4). Next, the average titer value for three animals was calculated.

### 2.12. Data Analysis

The “Statistica 13.0” program was used for data analysis. Cumulative probabilities of success were assessed using Kaplan–Meier survival curves, and the log-rank test was used for group comparisons. Comparisons of viral titers were performed using a single-factor analysis of variance (ANOVA), while pairwise comparisons between the groups were performed using Tukey’s HSD test. The differences between the groups were considered statistically significant if the *p*-value did not exceed 0.05.

## 3. Results

### 3.1. Constructing the Variants of the Epitope Vaccine

The epitopes were selected from the major proteins of influenza virus type A (subtypes H1 and H3) and influenza type B because they are the main human pathogens. T-cell epitopes were selected from the NP and M1 proteins, and B-cell epitopes were selected from the hemagglutinin (HA) protein, which is a target for neutralizing antibodies [35]. Epitopes were selected from the IEDB database based on their proven immunogenicity according to the literature, as well as on the basis of the conservation criteria (2^S^ < 1.3, the selection algorithm developed is in press, see methods). Fifteen conserved and immunogenic epitopes were selected from 992 epitopes (Table 1).

The selected epitopes were joined into polypeptides k1, k2 and k5 (Table 2) using a linker (a proline residue). In the polypeptides, each epitope was repeated 3 times [52]. Polypeptides k1 and k5 contained B-cell and CD4+ T-cell epitopes, and polypeptide k2 contained only T-cell epitopes.

Polypeptides with B-cell epitopes must interact with B-cell clones in a local lymph node to trigger an antibody immune response against them. In fact, these epitopes should be located on the surface of the polypeptides because they are recognized by immunoglobulins. Two variants of the constructs with different order of equal set of B-cell epitopes were made. The epitopes in the k1 construct were grouped based on their hydrophobicity: the N-terminus contained hydrophobic epitopes (more than 50% hydrophobic amino acid residues), and the C-terminus contained hydrophilic epitopes (more than 50% hydrophilic amino acid residues). In contrast to the k1 construct, the k5 construct used alternating hydrophobic and hydrophilic epitopes order (hydrophobic-hydrophilic-hydrophobic-…). Additionally, the CD4+ epitopes of the HA protein were added to the constructs with B-cell epitopes (k1, k5) because B cells need to receive a proliferation signal from CD4+ cells during the immune response.

The epitopes in the k2 construct were joined without several variations because we adhered to the opinion that the conformation is not important for epitope processing and presentation in classes I and II MHC molecules.

### 3.2. Constructing Reference Constructs Variants for Comparison

For comparison, we selected antigens from vaccines well studied in the literature whose immunogenicity, protection and, most importantly, universality were shown. We were interested in vaccines that use different antigens and stimulate different branches of the immune response. Genes encoding antigens from these vaccines were inserted into MVA to compare immunogenicity and protective efficacy with our vaccine variants under the same delivery conditions. Since we selected B-cell epitopes from the HA stem, we needed a comparator variant that induces antibodies against the HA stem. Impagliazzo et al. [11] constructed a stable trimer of the HA stem (hlHA) and showed that it stimulated the production of cross-reactive antibodies in mice, protecting against a wide range of strains. Moreover, our vaccine contained T-cell epitopes from the conserved main internal proteins NP and M1, so it was necessary to compare it with a vaccine based on the whole NP and M1 proteins. For this purpose, we selected the MVA-NP+M1 (Invictus) vaccine, which contains the NP and M1 antigens as part of the MVA vector and is in Phase 2 clinical trials [12,13]. Finally, since our experimental vaccine is an epitope-based vaccine, it was necessary to compare it with an epitope-based vaccine, and the M-001 vaccine, which is in Phase 3 clinical trials, was chosen [14,53]. To compare our vaccine variants with the reference constructs, it was important to create the same delivery conditions. In this regard, the coding sequences of the antigens in the control vaccines were taken from open sources (mini-HA #4900 [11], MVA-NP+M1 [54], M001 [52]), synthesized and inserted into the MVA vector.

### 3.3. Obtaining Recombinant MVA and Checking Antigen Expression

The synthesized DNA sequences encoding k1, k2, k5, M001, NP+M1 and hlHA were cloned downstream of vaccinia virus promoter 7.5 in shuttle vectors, which were used to obtain rMVA.

**Figure 1:**
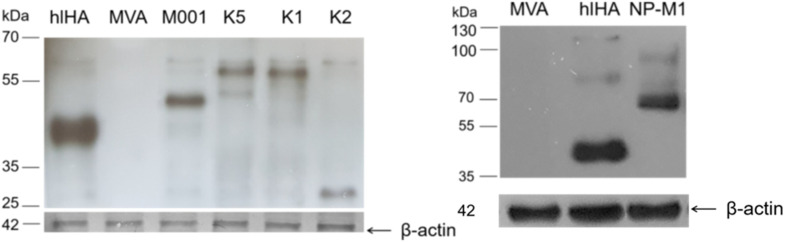
Western blot analysis of antigen expression in BHK-21 cells infected with rMVA-hlHA, rMVA-M001, rMVA-k5, rMVA-k1, rMVA-k2, rMVA-NP-M1 or wild-type MVA viruses. After 24 h of infection or transfection, cell lysates were obtained, separated using 12% SDS–PAGE under reducing conditions, transferred to a nitrocellulose membrane and detected using antibodies against 6xHis or antibodies specific to beta-actin. kDa is a protein molecular weight marker. Wild-type MVA was used as a negative control for rMVA-infected cells. Protein weights (kDa): k1—45.3, k2—31.1, k5—45.3, hlHA—42, NP+M1—84.9, M001—52.5.

Antigen expression in BHK-21 cells infected with one of the recombinant viruses, rMVA-k1, rMVA-k2, rMVA-k5, rMVA-M001, rMVA-NP+M1 or rMVA-hlHA was evaluated via Western blotting. According to the results of immunoblotting (Figure 1), antigen expression was observed in all of the studied samples, and the proteins were the expected sizes (Table S1). However, antigens k1, k2 or k5 was not found in supernatants of the BHK cells.

### 3.4. Study of the Antibody Response to Different Strains of Influenza Virus Types A and B

The resulting rMVAs were used to vaccinate mice. Six experimental groups of mice were used, as well as a control group of naive (mock) mice and a control group (empty MVA) of mice that were immunized with wt MVA (Table 3). Mice from group 1 (rMVA-k1-k2) were injected twice with a 1:1 mixture of rMVA containing the k1 epitope variant (rMVA-k1) and rMVA containing k2 epitope variant (rMVA-k2). The second group (rMVA-k5-k2) was injected twice with a 1:1 mixture of rMVA containing the k5 (rMVA-k5) and k2 (rMVA-k2) epitope variants. The remaining groups were injected twice with rMVA-M001-, rMVA-NP+M1- or rMVA-hlHA-containing antigens from known vaccines.

After the first and second immunizations, antibodies response to A/California/07/09 (H1N1), A/Aichi/2/68 (H3N2), B/Brisbane /60/2008 and B/Wisconsin/1/2010 in mice was evaluated using HAI and the neutralization tests. After the first immunization in all groups of animals, neither the HAI test nor the neutralization detected antibody production against any of the four influenza A and B viruses studied. However, after the second immunization, the HAI test found antibodies against the H1-subtype strain in animals in the group vaccinated with rMVA-k1-k2. In mice vaccinated with rMVA-M001 or rMVA-hlHA, antibodies against the H3-subtype strain were detected via both the HAI test and neutralization (Table S2). No antibodies were detected in the animals from the other groups.

### 3.5. Protection of Mice against Challenge Infection

**Figure 2:**
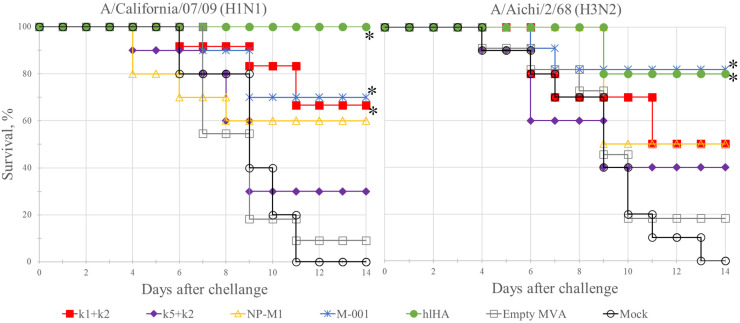
Kaplan–Meier survival analysis for results of infecting animals (10 to 12 mice per infected group) with the A/California/04/09 (H1N1) or A/Aichi/2/68 (H3N2) viruses after immunization with rMVAs expressing influenza virus antigens, or with wt MVA, or PBS (Mock). A statistically significant difference (*p*-value < 0.05 according to the Mantel–Cox log-rank test) relative to the controls is shown with asterisk.

We studied the protective properties of the epitope variants of the vaccines and the reference constructs against viral challenge in a mouse model. On the 14th day after the second immunization, the mice in each group were divided into two subgroups, one of which was infected with H1N1 strain A/California/07/2009 and the other with H3N2 subtype A/Aichi/2/68. Mice were observed for 14 days after infection (Figure 2).

Mock mice infected with the A/California/07/09 (H1N1) or A/Aichi/2/68 (H3N2) virus died within 6 to 11 days and within 4 to 13 days, respectively, with 100% mortality; the average lifetimes after infection in these groups were 8 and 7.8 days, respectively. Similar mortality rates (91% for H1N1 and 82% for H3N2) and average lifetimes (7.9 and 8.7 days) were observed in the group (Empty MVA) vaccinated with wt MVA that did not contain influenza virus antigens. Among the experimental variants, the mixture of rMVA-k5 and rMVA-k2 had the weakest protective activity. The death rate of the mice in this group rMVA-k5-k2 was 70% for H1N1 and 60% for H3N2, and the average lifetime was close to the lifetime of the animals in the control groups (9.2 for H1N1 and 9.4 for H3N2). Statistically significant increases in survival relative to the controls (*p*-value < 0.05) were observed in the group rMVA-k1-k2 (34% mortality for H1N1 and 50% mortality for H3N2). Additionally, significant differences relative to the controls (*p*-value < 0.05) were observed in the groups vaccinated with the reference constructs rMVA-NP+M1, rMVA-M001 and rMVA-hlHA (Figure 2).

**Figure A1:**
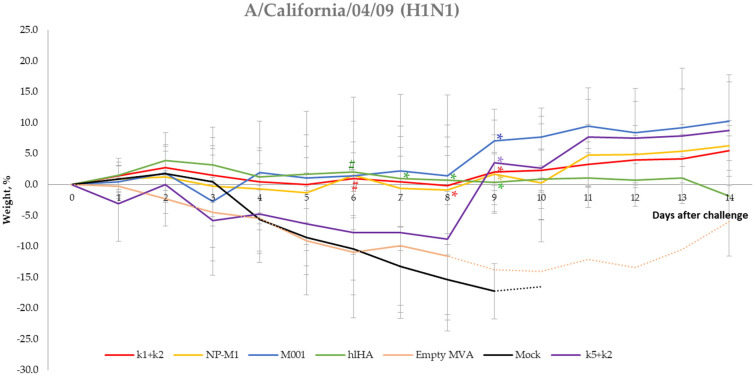
Loss of body weight of mice immunized with rMVAs expressing influenza virus antigens, or with wt MVA, or PBS after virus challenge with the H1N1 subtype virus strain A/California/04/09. The dotted line indicates that there are less than four mice. Significance codes (*p*-value < 0.05): * compared to Mock group, # compared to Empty MVA group.

**Figure A2:**
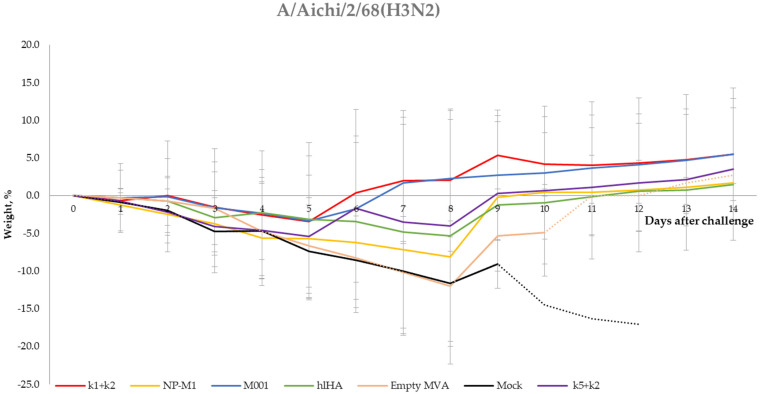
Loss of body weight of mice immunized with rMVAs expressing influenza virus antigens or with wt MVA or PBS after virus challenge with the H3N2 subtype virus strain A/Aichi/2/68. The dotted line indicates that there are less than four mice.

In addition, body weight of mice was determined daily as an indicator of disease (Figure A1 and Figure A2). Statistically significant differences in body weight loss after virus challenge with the strain A/California/04/09 (H1N1) were found for several groups compared to control groups (Figure A1). Although the protection of mice against challenge with A/Aichi/2/68 (H3N2) infection in several groups was observed, there were no differences in body weight loss (Figure A2).

### 3.6. Viral Titer in the Lungs of Mice after Infection with H1N1 and H3N2 Influenza Virus Subtypes

The viral titer in the lungs of the mice was determined on Day 4 after infection. The viral titers in the lungs (log10) were highest in the control groups of unvaccinated mice (Mock) and mice vaccinated with wt MVA (Empty MVA); they were 7.5 ± 0.5 and 7 ± 1.8 for influenza A/California/04/09 (H1N1) and 5.33 ± 0.58 and 5.33 ± 0.58 for A/Aichi/2/68 (H3N2), respectively.

**Figure 3:**
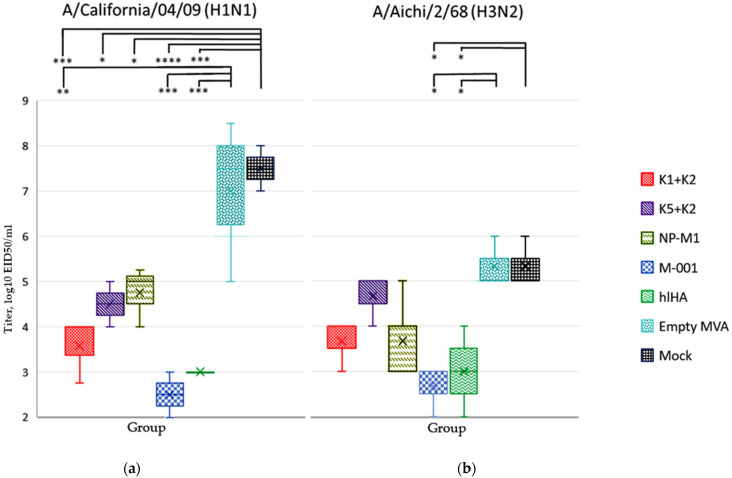
The viral titers (log_10_ EID_50_/_mL_) in the lungs of mice immunized with rMVAs expressing influenza virus antigens or with wt MVA or PBS after infection with influenza viruses (**a**) A/California/04/09 (H1N1) or (**b**) A/Aichi/2/68 (H3N2). The median (*n* = 3) is shown by a bold line inside the boxes. The boxes themselves show the boundaries of the lower and upper quartiles of the distribution covering 50% of the samples, and the ends of the whiskers are the edges of a statistically significant sample (99.3%). The survival rate in each group is indicated at the top. The statistical significance of the differences was assessed using Tukey’s test. Significance: * *p*-value < 0.05, ** *p*-value < 0.01, *** *p*-value < 0.001, **** *p*-value < 0.0001.

When infected with H1N1, the mice in all the experimental groups showed viral titers in the lungs that were significantly lower than those in the lungs of naive (mock) mice (Figure 3). The greatest difference with respect to the mock was observed in the groups rMVA-k1-k2, rMVA-M001, and rMVA-hlHA; in these groups, the titer was 3.9–5 orders of magnitude lower (*p*-value < 0.001). A smaller difference (2.5–3 orders of value) relative to the mock group was observed in the rMVA-k5-k2 and rMVA-NP+M1 groups (*p*-value < 0.05). In addition, when infected with H1N1, there was a statistically significant (*p*-value < 0.01) decrease in the titer in the lungs by 3.4–4.5 orders of magnitude in the groups rMVA-k1-k2, rMVA-M001 and rMVA-hlHA relative to the group vaccinated with wt MVA. This fact suggests that a statistically significant decrease in the titer of the H1N1 virus was caused by immunization with influenza antigens k1 + k2, M001 or hlHA.

In the case of H3N2 infection, the viral titer in mice vaccinated with influenza antigens also decreased in all groups; however, this difference was statistically significant (*p*-value < 0.05) only for the groups rMVA-M001 or rMVA-hlHA.

### 3.7. Assessment of the Breadth of Immunogenicity and Protection of the rMVA-k1-k2 Vaccine Variant

Since the greatest protective effect among our experimental variants of the epitope vaccine was achieved when mice were vaccinated with a mixture of rMVA carrying the k1 and k2 epitope constructs (rMVA-k1-k2), this variant was studied in more detail in further experiments. Thus, the immunogenic response to and protection conferred by the rMVA-k1-k2 vaccine variant were studied in mice using eight strains of different subtypes of A- and B-type influenza viruses (Table 4). For comparison, a seasonal Flu-M vaccine (2019–2020 season) was used that included strains of the H1N1 and H3N2 subtypes and type B (see methods, Section 2.2). Two experimental groups of mice were used (the rMVA-k1-k2 group and the seasonal vaccine group), as well as one control group of mock mice. Immunization was conducted using the same scheme as in the previous experiment (see methods, Section 2.7).

The assessment of humoral immunity using the HAI test with 8 viral strains showed that after the first immunization, antibodies were detected only in mice vaccinated with the seasonal vaccine; antibodies to the A/California/07/09 virus (H1N1) were detected in titers 40, 80, 80 and 80 (Table S3). After the second immunization in the group rMVA-k1-k2, antibody production was detected in titers from 1:20 to 1:40 against 4 influenza A viruses: A/California/07/2009 (H1N1), A/Puerto Rico/8/34 (H1N1), A/Aichi/2/68 (H3N2) and A/Chicken/Kurgan (H5N1). In the group vaccinated with the seasonal vaccine, an antibody titer >1:40 was detected for two influenza viruses, A/California/07/2009 (H1N1) and B/Colorado/06/2017, while in the same group, the production of antibodies against four other viruses studied was not detected (Table 4, Tables S3 and S4).

**Figure 4:**
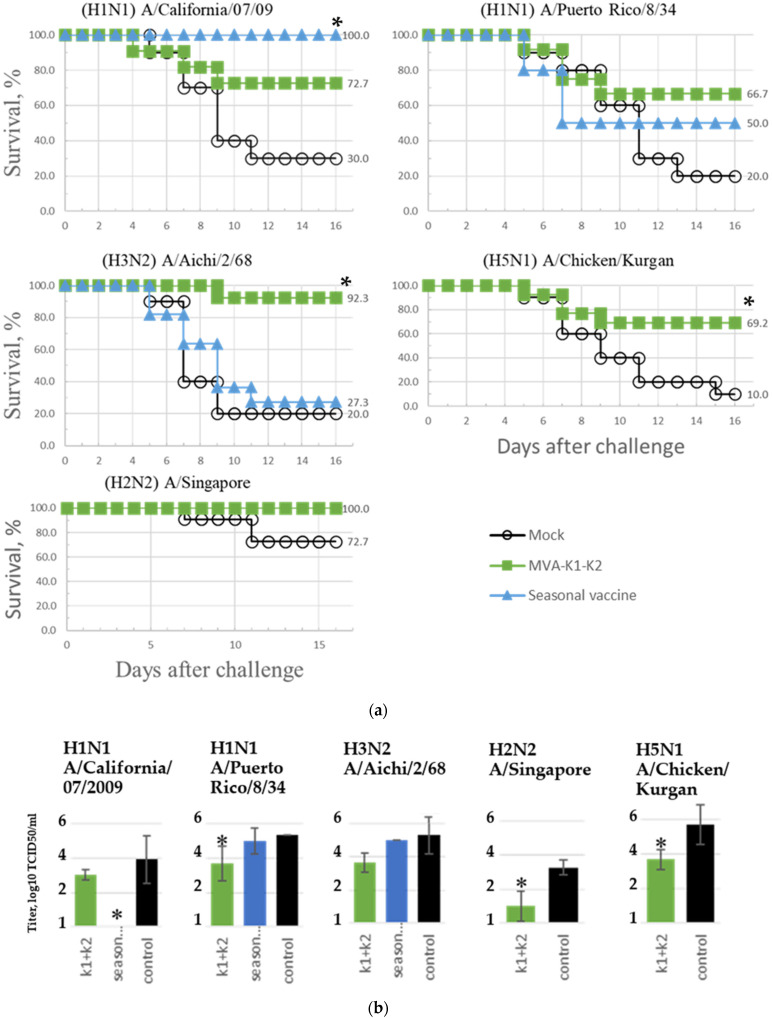
Results of infecting animals with different viral strains after immunization with rMVA-k1-k2, seasonal vaccine or PBS (Mock). Significance compared to control (mock): * *p*-value < 0.05. (**a**) Kaplan–Meier survival analysis (10 to 14 mice per infected group). (**b**) Viral titers (log_10_ TCID_50_/_mL_) in the lungs, mean value (*n* = 3).

The protection of the rMVA-k1-k2 vaccine variant against five influenza A strains and the seasonal vaccine against three strains was evaluated based on the survival rate of mice. The results are presented in Figure 4a and in Table 4. When animals from the control group were infected with influenza A viruses, the mortality rate reached 70–90%, with the exception of infection with the A/Singapore virus (H2N2), which led to a mortality rate of no more than 30%. In the group rMVA-k1-k2, 67–100% of the mice infected with various strains survived. Similar protection against strains of different subtypes indicates the universal properties of the vaccine. For the seasonal vaccine, 100% protection was observed only against strain A/California/07/09; for the older strain of the same subtype, A/Puerto Rico/8/34, protection fell to 50%, and when infected with A/Aichi/2/68 of the H3N2 subtype, a 27% survival rate was observed.

Additionally, in each group, the viral titer in the lungs was determined. In mice that received a seasonal vaccine, the virus was not detected in the lungs when infected with A/California/07/09; in the other cases, the viral titer was at the control level. At the same time, the viral titer in the lungs of mice vaccinated with rMVA-k1-k2 vaccine variant decreased by 0.9–2.25 orders of magnitude compared to the negative control, but this difference was significant (*p*-value < 0.05) only for strains A/Puerto Rico/8/34 (H1N1), A/Chicken/Kurgan (H5N1), and A/Singapore (H2N2) (Figure 4b).

## 4. Discussion

In this paper, we developed an experimental universal epitope-based flu vaccine using the MVA vector and our own algorithms to select the epitopes. We studied the immunogenicity and protective effectiveness of our vaccine against a wide range of influenza virus strains in a mouse model. The rMVA-k1-k2 vaccine consists of an equal mixture of two recombinant MVAs (modified smallpox vaccinia viruses of the Ankara strain) whose genomes contain cassettes for the expression of the k1 and k2 epitope constructs, which we created. Construct k1 encodes a polypeptide carrying B-cell and CD4+ T-cell epitopes, and construct k2 is a polypeptide carrying CD4+ and CD8+ T-cell epitopes. The epitopes were selected from the conserved regions of the main immunogenic proteins of the influenza virus, HA, NP and M1, while other properties of the selected epitopes, such as allergenicity and the ability to induce autoimmune antibodies [55], were not analyzed in our study.

Although epitope-based vaccines have many advantages, there are some problems in generating an efficient epitope-based vaccine. First of all, it is known that peptide vaccines have weak immunogenicity. To solve this problem, we used MVA vector to increase immunogenicity because it mediates recruitment and activation of immune cells.

Another limitation is that only linear epitopes can be used to induce a humoral response. The formation of the antibodies to target epitopes depends on the correct location of these epitopes in the polypeptide. Thus, the optimal structure is difficult to predict. During the creation of the vaccine, two variants of the epitope polypeptide aimed at the development of a humoral immune response were tested: k1 and k5. The B-cell and CD4+ T-cell epitopes had the same composition, but they were placed in different orders. In our opinion, the order was important because it was necessary to create and select an antigen with the appropriate presentation of B-cell epitopes on the surface of the protein molecule. A comparison of the protective effects of the rMVA-k1-k2 and rMVA-k5 + k2 vaccine variants showed that both the survival rate and viral titer when infected with H1N1 or H3N2 influenza viral strains were better for the rMVA-k1-k2 variant than for rMVA-k5 + k2, although the differences did not reach statistical significance. The obtained data allow us to speculate that in the case of the k1 antigen, the presentation of epitopes was more successful; thus, the principle used in k1 of grouping hydrophobic epitopes at the N-terminus and hydrophilic epitopes at the C-terminus turned out to be a better strategy than that used for k5, which alternated hydrophilic and hydrophobic epitopes.

To assess the immunogenic and protective properties of the developed vaccine, we designed reference constructs based on the most promising universal vaccines from the literature. The antigen-encoding sequences from these vaccines were integrated into the MVA vector. The obtained rMVA provided a comparison under the same conditions as all the antigens studied in the present work. As one of the controls, the Invictus vaccine [54] containing the full-length NP and M1 proteins as part of the MVA vector was used. Notably, this reference construct is as similar as possible to the original in view of a similar delivery. As the developers of this vaccine have shown in clinical trials, the antigen delivered using the MVA vector does not cause the production of neutralizing antibodies but can effectively induce a T-cell response [12,13]. Thus, in our experiment, it served as a control of the effectiveness of the protection provided by T-cell immunity. Another reference control was a conserved stabilized fragment of the HA stem (hlHA, headless HA), which causes the production of neutralizing antibodies to heterologous influenza strains (H1N1, H5N1) [11]. The last reference control was the M-001 epitope vaccine, which used the same approach as that used to create our polypeptide but differed in epitope composition and delivery method. The M-001 epitope polypeptide is capable of stimulating both branches of the immune response [14,36,53]. It should be noted that there was a limitation in our comparison strategy because we did not study how the delivery method could affect the immunogenicity of the reference antigens.

According to the results of the comparison, the humoral immunity caused by all of the studied vaccines was low (Table S2). In some cases, HAI titers was observed without MN titers for rMVA-k1-k2. This can be explained by the fact that agreement of nominal titers is moderate [56]. Thus, MN titers could be below the detection threshold level. Nevertheless, all of the studied vaccines demonstrated a protective effect. This result may indicate that the T-cell response plays an important role in antiviral protection. This idea is confirmed by a study of the Invictus vaccine (MVA-NP+M1), which demonstrated the generation of a T-cell response in humans after the introduction of the vaccine [12,13]. In addition, protective immunity could be due to the influence of non-neutralizing antibodies that are not detected by the HAI and neutralization tests but can contribute to protection due to the mechanisms of antibody-dependent phagocytosis, antibody-dependent cellular cytotoxicity and complement-dependent cytotoxicity. In the future, we plan to assess the T-cell response and antibody-dependent cytotoxic effect. Besides, the protective efficacy of the vaccine against strains of virus B will be evaluated in future studies.

Notably, the level of protection in the group of mice vaccinated with rMVA-k1-k2 vaccine was comparable to that in the groups vaccinated with the reference constructs rMVA-hlHA, rMVA-M001 and rMVA-NP+M1. Since M001 (recombinant protein) and MVA-NP+M1 (Invictus) are currently being successfully tested in clinical trials, the vaccine we have created is highly promising.

The study of the protection conferred by rMVA-k1-k2 vaccine against serologically distant influenza strains in comparison with a seasonal vaccine is of the greatest interest. When the strain in the seasonal vaccine coincided with the influenza strain used for infection (in the case of A/California/07/2009 (H1N1)), as expected, 100% protection was observed. When immunized mice were infected with strains with more-distant antigenic characteristics, the protection provided by the seasonal vaccine was significantly reduced. A 50% survival rate was observed for mice infected with A/Puerto Rico/8/34 (H1N1), and only a 27% survival rate was observed for mice infected with the A/Aichi/2/68 subtype (H3N2 strain) (with 20% survival in the control group). At the same time, the rMVA-k1-k2 vaccine provided protection for 67% to 92% of animals against H1N1- and H3N2-subtype viruses. When infected with the A/Singapore strain of the H2N2 subtype, 100% protection was observed, but the model in this case was not lethal. However, there was a decrease in the viral titer in the lungs. In addition, it is important to note that vaccination with rMVA-k1-k2 vaccine protected 70% of mice from the lethal course of the avian influenza A/chicken/Kurgan strain (H5N1 subtype). Protection against these strains is extremely important since seasonal vaccines do not induce protection against them, but they are considered potentially pandemic [2,3,57,58,59].

Thus, we have developed an epitope-based vaccine that confers protection against a wide range of H1N1, H2N2, H3N2 and H5N1 subtypes and is promising for further research on its use as a universal influenza vaccine.

## Figures and Tables

**Table 1:** Conserved immunogenic influenza virus epitopes selected from the IEDB database.

No.	Protein	Position	Sequence	Type/Subtype (Conservation)	Immune Response Type	References
1	HA	124–136	SVSSFERFEIFPK	A/H1 (100%)	B cell response	[36]
2	HA	18–32	DTLCIGYHANNSTDT	A/H1 (100%)	B cell response	[37,38]
3	HA	345–355	GLFGAIAGFIE	A/all (100%) and B (90%)	B cell response	[39,40]
4	HA	411–427	KEFSEVEGRIQDLEKYV	A/H3 (100%)	B cell response	[39,41]
5	HA	247–256	PNQTEDGGLP	B (51%)	B cell response	[42]
6	HA	247–256	PDQTEDGGLP	B (49%)	B cell response	[42]
7	HA	299–307	KGSLPLIGE	B (100%)	B cell response	[42]
8	HA	354–372	PAKLIKERGFFGAIAGFLE	B (100%)	B cell response	[43]
9	HA	318–332	GKCPKYVKSTKLRLATGLRN	A/H1 (74%)	Th response	[44]
10	HA	343–359	SRGLFGAIAGFIEGGWT	A/H1 (100%)	B cell + Th responses	[45]
11	NP	205–229	NFWRGENGRKTRSAYERMCNILKGK	A (92%)	Th + CTL responses	[46,47]
12	NP	37–54	GRFYIQMCTELKLSDYEG	A (100%)	CTL response	[48]
13	M1	180–197	VLASTTAKAMEQMAGSSE	A (100%)	Th response	[49]
14	M1	58–66	GILGFVFTL	A (100%)	CTL response	[50]
15	NP	85–94	KLGEFYNQM	B (100%)	CTL response	[51]

**Table 2:** The orders of the epitopes in the k1, k2 and k5 constructs. There are no epitopes 3 and 6 from Table 1 in k1 or k5 constructs because epitope 3 is a part of epitope 10, and epitope 6 is a part of 5′ (i.e., 5′ means the 5-6-5 epitope chain).

Name	Order of Epitopes ^1^	Immune Response Type
k1	8-1-10-7-2-9-4-5′ ^2^	B cell and Th responses
k2	11-12-13-14-15	Th and CTL responses
k5	8-1-9-10-5′-7-4-2	B cell and Th responses

^1^ The numbering is given in accordance with Table 1. ^2^ Means that 5′ is the 5-6-5 chain.

**Table 3:** Groups of mice with immunization scheme.

#	Group Name	Number of Mice	First Immunization	Second Immunization
1	rMVA-k1-k2	28	rMVA-k1 + rMVA-k2	rMVA-k1 + rMVA-k2
2	rMVA-k5-k2	26	rMVA-k5 + rMVA-k2	rMVA-k5 + rMVA-k2
3	rMVA-NP+M1	26	rMVA-NP+M1	rMVA-NP+M1
4	rMVA-M001	27	rMVA-M001	rMVA-M001
5	rMVA-hlHA	26	rMVA-hlHA	rMVA-hlHA
6	Empty MVA	28	wt MVA	wt MVA
7	Mock	26	PBS	PBS

**Table 4:** HAI titer against 8 strains 14 days after the second immunization.

#	Strain	Groups Vaccinated with		Mock Group
		rMVA-k1-k2	Seasonal Vaccine (2019–2020)	
1	A/California/07/2009 (H1N1)	30	120	<10
2	A/Puerto Rico/8/34 (H1N1)	27	<10	<10
3	A/Aichi/2/68 (H3N2)	27	<10	<10
4	A/Texas/50/2012 (H3N2)	20	-	<10
5	A/Singapore (H2N2)	<10	-	<10
6	A/Chicken/Kurgan (H5N1)	40	<10	<10
7	B/Colorado/06/2017	<10	80	<10
8	B/Phuket/3073/2013	<10	<10	<10

## Data Availability

The data will be available upon reasonable request to the corresponding authors.

## Supplementary Materials

The following are available online at https://www.mdpi.com/article/10.3390/vaccines10040534/s1, Table S1: Characteristics of the constructions studied in the work, Table S2: Formation of antibodies after the second immunization against four influenza A and B viruses, detected in HAI and neutralization tests, Table S3: Formation of antibodies after the first immunization against six influenza A viruses and two group B viruses detected in HAI test, Table S4: Formation of antibodies after the second immunization against six influenza A viruses and two group B viruses detected in HAI test.

## Appendix A

**Table A1:** The following viruses were used to stimulate influenza infection in mice.

#	Strain	Source
1	A/California/07/2009 (pdm H1N1 2009) adapted to mice	WHO
2	A/Puerto Rico/8/34 (H1N1)	Smorodintsev Research Institute of Influenza
3	A/Chicken/Kurgan (H5N1)	Smorodintsev Research Institute of Influenza
4	A/Singapore (H2N2)	Smorodintsev Research Institute of Influenza
5	A/Aichi/2/68 (H3N2) adapted to mice	The National Research Center for Epidemiology and Microbiology named after the Honorary Academician N.F. Gamaleya of the Ministry of Health of the Russian Federation

**Table A2:** The following viruses were used to perform the hemagglutination inhibition (HAI) test and neutralization test.

#	Strain	Source
1	A/California/07/2009 (pdm H1N1 2009), adapted to mice	WHO
2	A/Puerto Rico/8/34 (H1N1)	Smorodintsev Research Institute of Influenza
3	A/Chicken/Kurgan (H5N1)	Smorodintsev Research Institute of Influenza
5	A/Aichi/2/68 (H3N2), adapted to mice	The National Research Center for Epidemiology and Microbiology named after the Honorary Academician N.F. Gamaleya of the Ministry of Health of the Russian Federation
6	A/Texas/50/2012 (H3N2)	CDC (Atlanta, GA, USA)
7	B/Brisbane/60/2008	CDC (Atlanta, GA, USA)
8	B/Colorado/06/2017	CDC (Atlanta, GA, USA)
9	B/Wisconsin/1/2010	CDC (Atlanta, GA, USA)
