# Supplementary material for: Development of a Universal Epitope-Based Influenza Vaccine and Evaluation of Its Effectiveness in Mice
Source: Vaccines (Basel). 2022 Mar 29;10(4):534. doi: 10.3390/vaccines10040534 (PMC9026828; doi:10.3390/vaccines10040534)
Supplement: Supplementary file 1 [file vaccines-10-00534-s001.zip › vaccines-1643374-supplementary.pdf]

vaccines

MDPI

Table S1. Characteristics of the constructions studied in the work. The calculations took into account the tags, but not the signal peptide (22 a.a., 2.3 kDa).

Name	Tags at C-end	Length, a.a.	Weight, kDa
	HA, Myc, 6xHis	415	45.3
k2	Myc, 6xHis	276	31.1
k5	HA, Myc, 6xHis	416	45.3
hlHA	Myc, 6xHis	292	32.9
NP+M1	6xHis	761	84.9
M-001	Myc, 6xHis	448	52.5

Table S2. Formation of antibodies after the second immunization against four influenza A and B viruses, detected in HAI and neutralization tests.

Group No.	Mouse No.	Viruses							
		A/California/07/09 (H1N1)		A/Aichi/2/68 (H3N2)		B/Brisbane/60/2008		B/Wisconsin/1/2010	
		HAI test	Neutralization test	HAI test	Neutralization test	HAI test	Neutralization test	HAI test	Neutralization test
MVA-k1-k2	1	40	20	<10	<10	<10	<10	<10	<10
	2	40	<10	<10	<10	<10	<10	<10	<10
	3	40	<10	<10	<10	<10	<10	<10	<10
	4	40	<10	<10	<10	<10	<10	<10	<10
MVA-k5-k2	1	<10	<10	<10	<10	<10	<10	<10	<10
	2	<10	<10	<10	<10	<10	<10	<10	<10
	3	<10	<10	<10	20	<10	<10	<10	<10
	4	<10	<10	<10	20	<10	<10	<10	<10
MVA-NP+M1	1	<10	<10	<10	<10	<10	<10	<10	<10
	2	<10	<10	<10	<10	<10	<10	<10	<10
	3	<10	<10	<10	<10	<10	<10	<10	<10
	4	<10	<10	<10	<10	<10	<10	<10	<10
MVA-M001	1	<10	<10	<10	<10	<10	<10	<10	<10
	2	<10	<10	40	40	<10	<10	<10	<10
	3	<10	<10	40	80	<10	<10	<10	<10
	4	<10	<10	40	40	<10	<10	<10	<10
MVA-hlHA	1	<10	<10	80	160	<10	<10	<10	<10
	2	<10	<10	40	40	<10	<10	<10	<10
	3	<10	<10	<10	<10	<10	<10	<10	<10
	4	<10	<10	40	40	<10	<10	<10	<10

Empty MVA	1	<10	<10	<10	<10	<10	<10	<10	<10
	2	<10	<10	<10	<10	<10	<10	<10	<10
	3	<10	<10	<10	<10	<10	<10	<10	<10
	4	<10	<10	<10	<10	<10	<10	<10	<10
Mock	1	<10	<10	<10	<10	<10	<10	<10	<10
	2	<10	<10	<10	<10	<10	<10	<10	<10
	3	<10	<10	<10	<10	<10	<10	<10	<10
	4	<10	<10	<10	<10	<10	<10	<10	<10

Table S3. Formation of antibodies after the first immunization against six influenza A viruses and two group B viruses detected in HAI test. Viruses: (I) A/California/07/09, (II) A/Puerto Rico/8/34, (III) A/Aichi/2/68, (IV) A/Texas/50 /2012, (V) A/Chicken/Kurgan, (VI) A/Singapore, (VII) B/Colorado/06/2017, (VIII) B/Phuket /3073/2013.

Group No.	Mouse No.	Viruses							
		(H1N1)		(H3N2)		H5N1	H2N2		
		I	II	III	IV	V	VI	VII	VIII
MVA-k1+k2	1	<10	<10	<10	<10	<10	<10	<10	<10
	2	<10	<10	<10	<10	<10	<10	<10	<10
	3	<10	<10	<10	<10	<10	<10	<10	<10
	4	<10	<10	<10	<10	<10	<10	<10	<10
Flu-M	1	40	<10	<10	n.i.*	<10	n.i.	<10	<10
	2	80	<10	<10	n.i.	<10	n.i.	<10	<10
	3	80	<10	<10	n.i.	<10	n.i.	<10	<10
	4	80	<10	<10	n.i.	<10	n.i.	<10	<10
Mock	1	<10	<10	<10	<10	<10	<10	<10	<10
	2	<10	<10	<10	<10	<10	<10	<10	<10
	3	<10	<10	<10	<10	<10	<10	<10	<10
	4	<10	<10	<10	<10	<10	<10	<10	<10

* n.i. – not investigated.

Table S4. Formation of antibodies after the second immunization against six influenza A viruses and two group B viruses detected in HAI test. Viruses: (I) A/California/07/09, (II) A/Puerto Rico/8/34, (III) A/Aichi/2/68, (IV) A/Texas/50 /2012, (V) A/Chicken/Kurgan, (VI) A/Singapore, (VII) B/Colorado/06/2017, (VIII) B/Phuket /3073/2013.

Group No.	Mouse No.	Viruses							
		(H1N1)		(H3N2)		H5N1	H2N2		
		I	II	III	IV	V	VI	VII	VIII
MVA-k1+k2	1	40	40	<10	<10	<10	<10	<10	<10
	2	40	<10	<10	<10	80	<10	<10	<10
	3	40	40	40	<10	<10	<10	<10	<10
	4	<10	<10	40	<10	40	<10	<10	<10
Flu-M	1	80	<10	<10	n.i. *	<10	n.i.	80	<10
	2	160	<10	<10	n.i.	<10	n.i.	80	<10
	3	160	<10	<10	n.i.	<10	n.i.	80	<10
	4	160	<10	<10	n.i.	<10	n.i.	0	<10
Mock	1	<10	<10	<10	<10	<10	<10	<10	<10
	2	<10	<10	<10	<10	<10	<10	<10	<10
	3	<10	<10	<10	<10	<10	<10	<10	<10
	4	<10	<10	<10	<10	<10	<10	<10	<10

* n.i.- not investigated.
